# Work is associated with a more robust rest-activity rhythm and high-intensity physical activity among older adults

**DOI:** 10.1007/s40520-025-03083-8

**Published:** 2025-06-28

**Authors:** Pin-Shiuan Lee, Yen-Ling Liu, Yi-Ling Chen, Wan-Ju Cheng

**Affiliations:** 1 National Center for Geriatrics and Welfare Research, National Health Research Institutes, 35 Keyan Road, Miaoli County, Miaoli County, Taiwan; 2 Department of Public Health, China Medical University, 100 Sec.1, Jingmao Rd, Taichung, Taiwan; 3 Department of Psychiatry, China Medical University Hospital, 2 Yude Road, Taichung, Taiwan

**Keywords:** Actigraphy, Sleep, Melatonin, Employment, Rest-activity rhythm

## Abstract

**Background:**

Work participation is a major element of active aging in aging societies. However, the impact of work on physical activity and rest-activity rhythm has not been well-studied in the older population.

**Aims:**

To investigate the association of work status with the distribution of physical activity and rest-activity rhythm.

**Methods:**

The study recruited 35 working and 72 non-working community-dwelling adults older than 60 years old. Biological rhythm was evaluated by dim light melatonin onset (DLMO). Activity distribution and rest-activity rhythm indicators were derived from 14-day actigraphy data, and differences between working and non-working groups were analyzed. The association of activity distribution and rest-activity rhythm indicators with mood symptom was examined.

**Results:**

Compared to non-working older adults, those who were working exhibited an earlier wake-up time (05:24 h vs. 06:11 h, *p* = 0.001) and higher levels of daytime activity (most active 10-hour activity count: 25605 vs. 16838, *p* < 0.001), but similar DLMO (20:20 h vs. 20:24 h, *p* = 0.914). Work is associated with a more robust rest-activity rhythm as assessed by interdaily stability (β = 0.18, *p* < 0.001) and autocorrelation coefficients (β = 0.09, *p* = 0.002). Regarding activity distribution, work is associated with high-intensity activity in shorter bouts, as shown by a lower Gini index (β = −0.04, *p* = 0.003) and a higher intensity gradient (β = 0.54, *p* < 0.001).

**Conclusions:**

Working and non-working older adults showed similar biological circadian rhythms, but working ones had a more robust rest-activity rhythm and higher levels and intensity of physical activity.

## Introduction

The concept of “active ageing” has been advocated by the World Health Organization since the 1990s [1], and promoting employment in the older population is expected to be facilitated through policy formulation. Employment in older adults not only increases society’s workforce but also provides opportunities for social participation, enhancing the capacity for independent living. Research has indicated that employment in the older population has been associated with a higher level of physical activity [2], whereas physical activity has shown significant mental health benefits [3]. While an association between employment in older ages and mood has remained inconclusive [4, 5], physical activity and rest-activity rhythm may be influencing factors in this relationship.

Disturbances in sleep-wake rhythms impair sleep, metabolism, and mental and physical health of older adults [6]. Numerous studies have revealed the association of late and irregular rest-wake rhythms with poor health [7]. In a population-based study, a reduction in relative amplitude (RA) was associated with an increased risk of lifetime major depressive disorder and lifetime bipolar disorder [8]. Older adults were observed to have an association between rhythm stability and brain white matter integrity [9], suggesting a relationship with cognitive functions. Despite their potential for exploring the physiological linkages of activity, sleep, and neuropsychiatric conditions, actigraphy data and studies regarding circadian rhythms among older adults have remained scarce in the literature.

Different types of physical activity have distinct effects on health. Conventional measurement consists of self-reported frequency, intensity, time, and type of physical activity. Numerous studies have shown that physical activities significantly improve sleep, enhance social-emotional function, and reduce frailty in older adults, while sedentary behavior may contribute to poor affective and physical feeling states or worsen sleep quality in older adults [10–12]. Recent research has proposed novel indicators regarding intensity distribution, activity accumulation, and temporal correlation and regularity [13]. Intensity distribution reflects the time spent across varying levels of activity intensity, providing insight into how active or sedentary a person is throughout the day. Activity accumulation, on the other hand, quantifies the distribution of activity bout durations over time. Both metrics describe the pattern of activity distribution beyond the total amount of activity. Temporal correlation and regularity assess how consistently the activity rhythm repeats every 24 h, reflecting the robustness of the rest-activity rhythm. These assessments have been applied to older populations (65 years and older) and have shown an association of age with daily living functions [14], balance and mobility [15, 16], and emotional functioning [17–19].

There exists a knowledge gap regarding how work affects the lifestyle of older adults and subsequently influences their mental well-being. Furthermore, relatively few studies have introduced objective indicators related to activity distribution and rest-activity rhythm in older populations. In this study, we aim to examine (1) the association of work status with activity distribution and rest-activity rhythm among older adults, and (2) the association of activity distribution and rest-activity rhythm with mood symptoms. We hypothesize that, compared to the non-working group, the working group would show (1) a higher regularity of rest-activity rhythm and (2) a higher distribution of high-intensity activity. In regard to mood, our hypothesis posits that a higher regularity of rest-activity rhythm and a higher distribution of high-intensity physical activity are associated with fewer depressive symptoms.

## Methods

### Participants and design

A prior sample size calculation was conducted using G*Power 3.1.9.7, with the alpha level set at 0.05, the power set at 0.95, and the effect size set at 0.8 for t tests. The results indicated that at least 84 participants were required. This study recruited healthy community-dwelling adults over 60 years old through advertisements on community billboards. Exclusion criteria were (1) serious neurological or psychiatric diagnoses, (2) physical illness or severe trauma preventing independent ambulation, or (3) diagnosis with sleep disorders or current use of hypnotics. We recruited 35 working individuals and 72 non-working older adults to participate in our two-week study. Work status was self-reported, and working was defined as having a paid job or being self-employed. Participants were instructed to wear the actigraphy device for 14 consecutive days and to complete sleep logs. Sleep and mood symptoms were assessed through questionnaires, and biological circadian rhythm was evaluated following an at-home saliva-sampling protocol.

### Self‑report questionnaire

Age and gender information were self-reported, and work status was categorized as either currently working or not working. Participants were also asked to provide details on the duration and frequency of weekly exercises, body height, body weight, and the presence of chronic diseases. The Pittsburgh Sleep Quality Index (PSQI) was used to assess sleep quality over the past month, and a composite score was derived [20]. The 5-item Brief Symptom Rating Scale (BSRS-5) was used to assess mood symptoms [21].

### Dim light melatonin onset

The salivary melatonin test for dim-light melatonin onset (DLMO) in older adults has been validated [22]. Participants provided saliva samples for melatonin in their home environment and were instructed to maintain clean lips, abstain from consuming caffeine, bananas, alcoholic beverages, or smoking cigarettes [23, 24]. Saliva samples were collected every 30 min from 18:00 until 1 h after habitual sleep time on the final study date, with participants refraining from food or drink for 10 min before collection. Fifteen minutes before saliva collection, participants rinsed their mouths with plain room-temperature water without brushing their teeth during the study period. Physical activities during the collection period were to be avoided. Subjects filled a test tube with 5 ml of saliva, and the samples were stored in a freezer. Salivary melatonin concentration was determined using a direct melatonin enzyme-linked immunosorbent assay (ELISA) kit. DLMO was determined based on the two standard deviation threshold method, which uses the average of the first three melatonin data points plus two standard deviations of the same three points [25].

### Actigraphic data collection and analysis

Participants were instructed to wear a MotionWatch 8 wrist actigraphy (MW8, CamNtech Inc, England, UK) on their non-dominant wrist and keep a sleep diary for 14 consecutive days. Activity counts were derived in 15-second epochs, and sleep scoring was aided by the sleep log. Sleep period and non-wear were manually scored using MotionWatch 8 software support (MotionWare v1.2.28) and assisted by the sleep log. Additionally, we identified the non-wear period by the longest time of continuous zeros between 07:00 and 12:00, and between 13:00 and 22:00 [26]. The non-wear period averaged 2.19% of the total recording period. The epoch-wide 24-h activity was used to generate indicators for rest-wake rhythm, namely the autocorrelation coefficient at a lag of 24 h (i.e., 1440 min), as well as estimated get-up and lie-down times in generalized additive models. Moderate to vigorous physical activity (MVPA) was identified as ≥ 562.5 activity counts per minute [27]. MVPA distribution was further analyzed based on the Gini index, power-law exponent α, and intensity gradient.

The Gini index measures the extent to which the distribution of physical activity among individuals deviates from a perfectly equal distribution [28, 29]. An index of 0 indicates that lengths of MVPA bouts were equally distributed. Conversely, an index of 1 indicated a largely unequal MVPA distribution. The power-law exponent α served as an index of activity accumulation [30], reflecting the distribution of MVPA bouts across all MVPA time. The association between bout length and density of bouts (activity counts) was plotted on a logarithmic scale. The power distribution of the bouts, estimated from the shape of the histogram, was characterized by the power-law exponent α. A larger power-law exponent α indicated an accumulation pattern with a greater proportion of shorter bouts at a specific intensity. The intensity gradient was calculated using R-package GGIR (version 2.10-1) [31, 32]. It represented the distribution of activity intensities across all MVPA times by describing the negative curvilinear relationship between activity intensities and the time accumulated at these intensities [33]. A lower intensity gradient (more negative) indicated a steeper drop as intensity increases, reflecting little time accumulated at higher intensities. All these indicators were calculated for MVPA using MATLAB (version 2023a, Mathworks).

For the evaluation of sleep-wake rhythm, the autocorrelation coefficient quantified the regularity of activity over a 24-h time-lagged data [34]. Additionally, generalized additive models (GAMs) [35, 36] were exploited to fit smoothed nonlinear curves to log-transformed aggregated actigraphy-derived activity measurements with the “mgcv” package (version 1.9-0) using R software (version 4.3.1). We fitted a GAM with 3 basis functions to the actigraphic data, with logarithmic activity as a smooth function of time by hour. Non-parametric analysis was utilized to generate (1) the intradaily variability (IV); (2) the interdaily stability (IS); (3) the time occurrence and corresponding activity counts of the most active 10-h period (M10) and of the least active 5-h period (L5); and (4) RA using MATLAB [37]. Cosinor analysis was performed using the cosinor and cosinor2 packages in R 4.3.0, assuming a known period and synchronized to a 24-h cycle. The corresponding mesor, amplitude, and acrophase were calculated [38]. 

### Statistical analyses

The differences in demographic characteristics, sleep, activity, rhythm indicators, and DLMO between the working and non-working groups were tested using the Mann-Whitney U test. We further examined the association of working conditions with DLMO and indicators for activity and rhythm using separate generalized linear regression models. The models were adjusted for age, sex, body mass index (BMI), chronic illness (hypertension, diabetes mellitus, cardiovascular disease, stroke, liver or kidney diseases), and BSRS-5 scores. We illustrated the 24-h activity distribution using GAM and cosinor analysis. The distribution of MVPA was depicted using data from one working and one non-working participant. To examine the association of activity and rhythm with mood symptoms, multivariate linear regression analysis was employed, wherein activity and rhythm indicators were mutually adjusted. All statistical analyses were performed using SAS 9.4 (SAS Institute, Cary, NC, USA).

## Results

### Sample characteristics

Table 1 shows the actigraphic characteristics of participants, with working (*N* = 35) and non-working (*N* = 72) older adults. Working older adults younger (67.49 vs. 71.07 years, *p* = 0.001), spent less weekly time exercising (106.26 vs. 282.85 min, *p* < 0.001), and had lower BSRS-5 scores (1.69 vs. 2.32, *p* = 0.077).

### Work status associated with activity and rhythm

Compared to non-working individuals, working older adults showed higher rhythm robustness, evidenced by higher IS (0.67 vs. 0.49, *p* < 0.001), lower IV (1.06 vs. 1.13, *p* = 0.04), and higher autocorrelation coefficient at lag 24 h (0.44 vs. 0.34, *p* = 0.001). The time of waking up (05:24 vs. 06:11, *p* = 0.001) and getting up (05:32 vs. 06:18, *p* < 0.001) were earlier among working older adults. However, there was no significant difference in DLMO between the two groups (20:20 vs. 20:24, *p* = 0.914). In regards to MVPA distribution, the working group demonstrated more evenly dispersed activity (Gini index: 0.15 vs. 0.20, *p* < 0.001), more time accumulated at higher intensity activities (intensity gradient: − 1.09 vs. -1.55, *p* = 0.004), and a greater proportion of shorter bouts of MVPA (power-law exponent α: -0.15 vs. -0.17, *p* = 0.008).

The association between work status and actigraphy-derived indicators after adjusting for age, gender, BMI, and BSRS-5 scores is shown in Table 2. Compared to non-working, working older is associated with an earlier get-up time, and higher M10, intensity gradient, auto-correlation coefficients, and mesor of the cosinor analysis. Additionally, work is associated with lower value in the Gini index and IV.

**Fig. 1:**
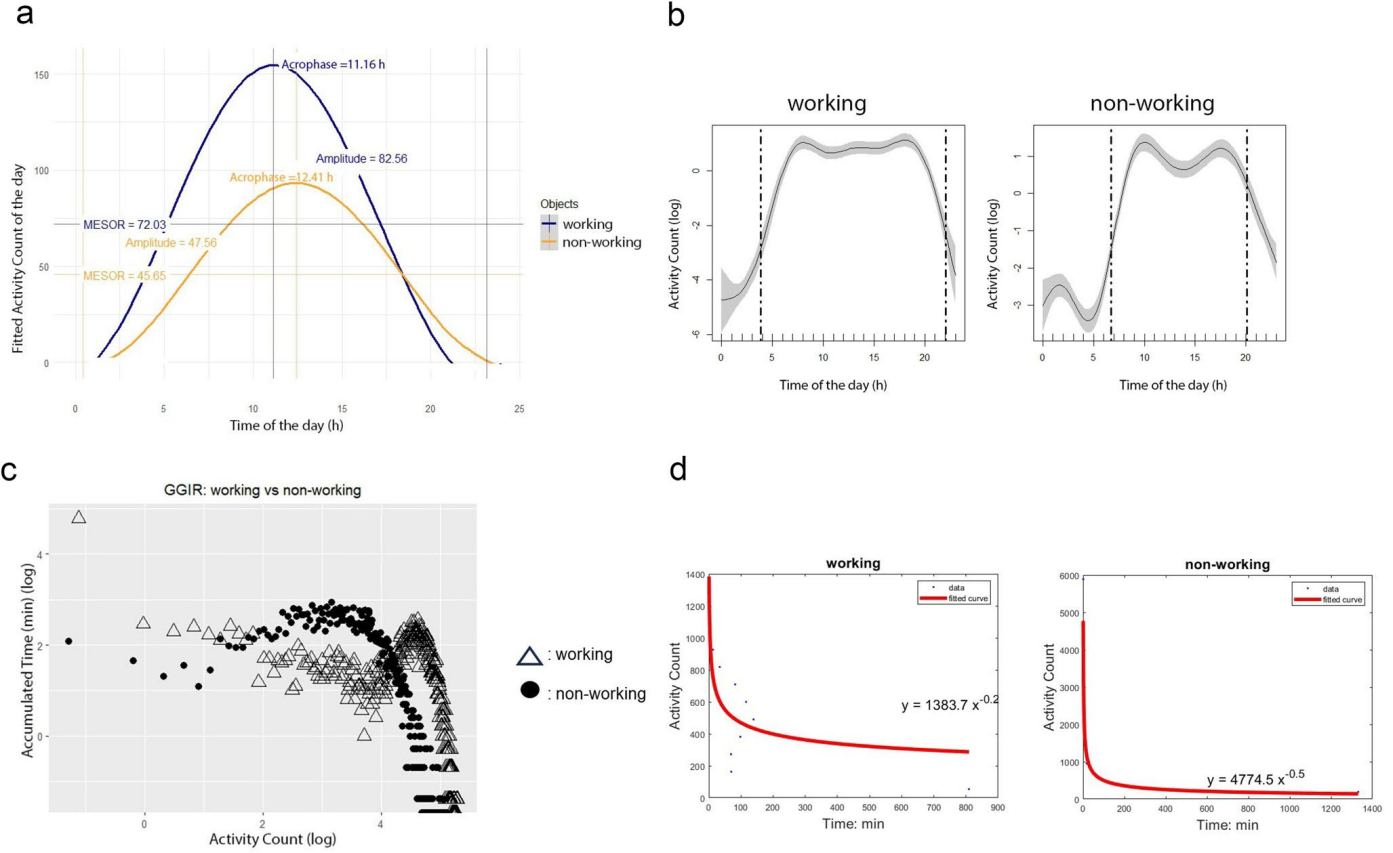
Visualizing 24-h activity distribution of working and non-working older adults. (**a**) Cosinor Curve: Circadian variation using cosinor analysis for a working adult (blue) and non-working adult (orange). (**b**) Activity of 24 h in generalized additive models (left dotted line = up-hour of GAM model; right dotted line = down-hour of GAM model). (**c**) Intensity gradient of moderate-to-vigorous physical activity distribution among working and non-working adults. (**d**) Power-law exponent α of moderate-to-vigorous physical activity distributions among working and non-working older adults.

Figure 1 provides an example of activity distribution for working and non-working older adults. In GAM models (Fig. 1A), working older adults showed an earlier get-up time, while non-working older adults displayed a dip in the afternoon, suggesting that the participant took daily naps. For working older adults, the cosinor curve showed higher mesor and amplitude (Fig. 1B), indicating greater activity and variance of activities within 24 h. In terms of MVPA distribution, working older adults demonstrated a higher intensity gradient, indicating more time accumulated at high intensities compared to their non-working counterparts (Fig. 1C). In Fig. 1D, the power-law exponent was higher among working older adults, suggesting a greater proportion of shorter bouts of MVPA.

### Activity and rhythm in relation to mood symptoms

We further predicted mood symptoms (BSRS-5 scores) with activity and rhythm variables in multivariate regression analysis (Table 3). Log-transformed L5 (*β* = -0.50, 95% CI = -0.88 to -0.12, *p* = 0.011) and RA (β = -14.23, 95% CI = -22.21 to -6.26, *p* = 0.001) were found to be negatively associated with BSRS-5 scores.

## Discussion

To the best of our knowledge, this study represents the first exploration of the differences in MVPA and rest-activity rhythm between working and non-working older adults. Working older adults exhibited an earlier and more regular rest-activity rhythm, along with a higher accumulation of time spent in higher intensity physical activities. Furthermore, rhythm regularity is associated with fewer depressive symptoms.

Our first novel finding indicates that working older adults exhibited an earlier and more regular rest-activity rhythm. The observed reduction in total sleep time among working older adults results from an earlier get-up time, despite having a bedtime similar to that of non-working older adults. This scheduling difference aligns with the concept that working older individuals maintain an early schedule compared to their retired counterparts, contributing to a reduced social jetlag [39]. While DLMO was consistently observed around 8 pm in our study, in line with previous observations in individuals older than 60 years [40], the working group exhibited a rest-activity rhythm more closely aligned with their biological rhythms. Conversely, non-working older adults experienced an increased social jetlag, which has been associated with both physical and mental health issues [39]. Importantly, a higher autocorrelation coefficient in the working group indicated a more stable activity rhythm compared to non-working older adults [41, 42]. This suggests that engagement in work may help maintain health in older adults, potentially through the establishment of a consistent daily schedule and the necessity to rise early.

The higher M10 value observed in working older adults suggests that, on average, they engage in higher activity counts during the daytime. Additionally, a lower GINI index and a higher intensity gradient suggest that more MVPA is dispersed in shorter bouts at higher intensities. In combination, working older adults exhibited more high-intensity activity and numerous bouts of activity during the daytime, while non-working adults showed fewer, longer bouts of activity with lower intensity. Physical activity has been linked to a reduced risk of major noncommunicable diseases [31, 43, 44]; additionally, structured exercise programs designed to push the body’s limits have been shown to enhance physiological markers such as muscular strength [45]. Studies on high-intensity multicomponent exercise programs for older adults have demonstrated positive effects, including reducing morning fatigue and preventing frailty [46–48]. Our findings suggest that non-working older adults may benefit from high-intensity physical activities to improve their health.

Nevertheless, it is intriguing to note that self-reported presence and duration of regular exercise were higher among non-working older adults compared to their working counterparts in this study. It is possible that the working group engages in MVPA during working hours rather than pursuing exercise during leisure time. Previous studies have indicated that MVPA at work and during leisure time may contribute differently to overall health outcomes [49, 50]. This divergence in activity patterns may contribute to the absence of a significant association between activity distribution and mood traits in our study. Furthermore, MVPA in this study was defined using a cutoff value validated in healthy adults over 55 years old [27]. However, the ability to perform MVPA varies by age and physical function, which refers to the capacity to perform activities of daily living and can be objectively measured using tests of grip strength, gait speed, and walking endurance [51, 52]. In future studies, assessing an individual’s physical function would help better delineate the health impact of activity patterns.

Our finding that a higher RA is associated with lower mood symptoms is consistent with previous studies [8]. However, the negative association between L5 activities and mood scores may seem contradictory to the assumption that better rest quality is linked to better mood. In our study, we utilized the 24-h mean profile of L5 over 14 days, which relates more with rest regularity than rest quality [53]. Therefore, our results highlight that a regular reset-activity rhythm is associated with fewer depressive symptoms, although the causal relationship may be reciprocal. The potential mechanism behind this phenomenon suggests that rhythm disturbance, such as night work, may reduce the quantity of extracellular serotonin in the synaptic gap by decreasing the methylation of the promoter of the serotonin transporter gene, leading to depression. We suggest that among older adults, the maintenance of a regular rest-activity rhythm in working older adults may contribute to enhanced emotion regulation.

This study has the strength of using objective actigraphy-derived indicators and salivary melatonin levels to simultaneously evaluate activity and circadian rhythm among working and non-working older adults. Compared to sleep log and objective questionnaires, actigraphy provides more detailed information on the rigor, time, and regularity of physical activity [54]. Nevertheless, this study has some limitations. First, the cross-sectional design hinders the ability to determine the directionality of the association between work and activity. For example, people with a more regular rest-activity rhythm may choose to work until older ages due to easier adaptation. Second, the type of work, e.g., sedentary or physically demanding work, has not been revealed by the participants. Different types of work may differ significantly in their physical activity during working hours. Additionally, we did not collect information regarding retirement payments for participants. Therefore, it is not clear whether the study participants’ decisions to work or not to work were influenced by their financial status, which may, in turn, affect their mood symptoms. Third, we estimated sleep based on actigraphy data. The absence of polysomnography for evaluating sleep quality and architecture is a limitation, as these factors may impact daytime activity and rest-activity rhythm, particularly among older individuals who are not working. Fourth, physical capacity was not assessed in this study. Although all participants were free from major illnesses and were independently mobile, differences in physical capacity could potentially confound the association between work and physical activity. Lastly, the sample size was relatively small, and the generalizability of the results is limited to community-dwelling older adults with fair health conditions.

Workforce aging presents challenges in maintaining physical performance, as documented declines in muscular strength, aerobic capacity, and physiological reserve can influence occupational performance trajectories [55]. Implementing evidence-based physical activity interventions and occupational health strategies represents a viable approach to preserving work ability despite advancing age. The substantial body of epidemiological evidence linking physical activity levels to mental health outcomes—particularly the consistent negative association with depressive symptoms—underscores the importance of movement behaviors in workplace health promotion. Previous research identifies reduced physical activity not merely because of depression [56, 57] but as a potential causal factor, positioning activity monitoring as a valuable preventive tool in occupational settings [58, 59]. Our research methodology employs actigraphy parameters, specifically M10 and relative amplitudes, as objective threshold metrics to further investigate relationships between physical activity profiles, mood states and sleep quality among older employees in the future study, with potential implications for workplace intervention design.

In summary, our results show that work may contribute to maintaining a regular and earlier rest-activity rhythm, accompanied by increased engagement in high-intensity physical activity. Given the known associations between reduced circadian activity rhythm robustness and both physical and mental health [60, 61], targeting rhythm regularity and phase becomes essential for health promotion among older adults [62]. Strategies such as adequate sleep hygiene, timed light exposure, and the use of melatonin have proven effective in restoring proper circadian patterns in the sleep-wake cycle. Furthermore, our study demonstrates the feasibility of incorporating these parameters for the evaluation of sleep and activity among older adults. In addition to addressing sleep, information on activity and rhythm provides valuable insights into promoting sleep health, enhancing emotional resilience, and improving overall quality of life in older adults.

**Table 1:** Differences in actigraphy indicators between working and non-working older adults, *p* values from Fisher’s exact test and Mann-Whitney tests

	Working (*N* = 35)		Non-working (*N* = 72)		*P*
Mean		(SD)	Mean	(SD)	
**Demographic Characteristics**					
Sex (female, N and %)	20	(57.14)	48	(66.67)	0.337
Age, years	67.49	(5.33)	71.07	(5.71)	0.001
Body mass index (kg/m^2^)	24.28	(3.58)	23.89	(3.41)	0.451
Chronic illness (N and %)	26	(74.29)	50	(69.44)	0.605
Regular exercise (N and %)	17	(48.57)	66	(91.67)	< 0.001
Weekly exercise time (min)	106.26	(129.01)	282.85	(322.97)	< 0.001
BSRS-5 score	1.69	(2.35)	2.32	(2.69)	0.077
**Rhythm Regularity**					
Interdaily stability	0.67	(0.18)	0.49	(0.11)	< 0.001
Intradaily variability	1.06	(0.17)	1.13	(0.18)	0.040
Relative amplitude	0.89	(0.08)	0.86	(0.10)	0.121
Autocorrelation coefficient	0.44	(0.14)	0.34	(0.13)	0.001
**Activity Counts**					
L5	880.57	(835.87)	939.24	(880.34)	0.937
M10	25605.11	(9346.47)	16838.22	(6539.59)	< 0.001
Mesor in cosinor analysis	47.14	(14.22)	38.06	(13.32)	< 0.001
**Time of Rhythm** (hh: mm)					
Lights-out	21:58	(1:27)	22:30	(1:14)	0.110
Fall-asleep	22:06	(2:14)	22:51	(1:11)	0.098
Wake-up	05:24	(0:53)	06:11	(1:06)	0.001
Get-up	05:32	(0:52)	06:18	(1:06)	< 0.001
Get-up hour in GAM	05:01	(1:31)	05:29	(0:56)	0.100
Lie-down hour in GAM	21:25	(1:14)	21:34	(1:25)	0.884
L5 start hour	23:17	(3:15)	23:25	(3:49)	0.410
M10 start hour	06:53	(2:57)	07:30	(3:04)	0.088
Acrophase in cosinor analysis	13:03	(1:53)	12:48	(1:39)	0.635
DLMO	20:20	(1:14)	20:24	(1:15)	0.914
**MVPA Distribution**					
Gini index	0.15	(0.05)	0.20	(0.07)	< 0.001
Intensity gradient	-1.09	(0.72)	-1.55	(0.62)	0.004
Power-law exponent α	-0.15	(0.08)	-0.17	(0.13)	0.008
**Sleep**					
Time in bed (hour)	7.60	(1.25)	7.80	(1.23)	0.344
Total sleep time (hour)	5.82	(1.44)	6.29	(1.07)	0.066
Wake after sleep onset (min)	67.80	(35.40)	61.20	(29.40)	0.437
Sleep onset latency (min)	30.00	(46.20)	21.00	(21.60)	0.944
PSQI score	3.91	(2.13)	4.93	(2.73)	0.075

*Abbreviations*: BSRS-5 = 5-item Brief Symptom Rating Scale; L5 = Average activity during the least active 5-hour period; M10 = Average activity during the most active 10-hour period; GAM = Generalized additive model; DLMO = Dim light melatonin onset; MVPA = Moderate to vigorous physical activity; PSQI = Pittsburgh Sleep Quality Index

**Table 2:** The association between work (reference: non-working) and actigraphy-derived indicators. Generalized linear models are adjusted for sex, age, body mass index, chronic illness, and 5-item Brief Symptom Rating Scale scores

Outcome variables	Estimate	95% confidence interval		*P*
**Rhythm Regularity**				
Interdaily stability	0.18	(0.12,	0.23)	< 0.001
Intradaily variability	-0.10	(-0.17,	-0.03)	0.008
Relative amplitude	0.02	(-0.02,	0.06)	0.251
Autocorrelation coefficient	0.09	(0.03,	0.14)	0.002
**Activity Counts**				
L5	-0.001	(-0.76,	0.76)	0.997
M10	0.40	(0.21,	0.58)	< 0.001
Mesor in cosinor analysis	9.46	(3.68,	15.25)	0.002
**Time of Rhythm**				
Lights-out	-0.77	(-1.31,	-0.23)	0.006
Get-Up	-0.82	(-1.27,	-0.38)	< 0.001
Get-up hour in GAM	-0.51	(-1.02,	-0.005)	0.048
Lie-down hour in GAM	0.08	(-0.51,	0.67)	0.797
L5 start hour	-0.29	(-1.89,	1.32)	0.726
M10 start hour	-0.72	(-2.03,	0.59)	0.280
Acrophase in cosinor analysis	0.12	(-0.62,	0.87)	0.745
DLMO	-0.15	(-0.70,	0.40)	0.586
**MVPA Distribution**				
Gini index	-0.04	(-0.07,	-0.01)	0.003
Intensity gradient	0.54	(0.26,	0.82)	< 0.001
Power-law exponent α	0.02	(-0.03,	0.07)	0.468

*Abbreviations*: L5 = Average activity during the least active 5-hour period; M10 = Average activity during the most active 10-hour period; GAM = Generalized additive model; DLMO = Dim light melatonin onset; MVPA = Moderate to Vigorous Physical Activity

**Table 3:** Linear regression model examining the association of selected rhythm and activity indicators and BSRS-5 scores, adjusted for age, sex, body mass index, and chronic illness

	BSRS-5			
Predicting variables	Estimate	95% CI		*P*
Working (reference: non-working)	11.77	(-3.75,	27.29)	0.135
**Demographic Characteristics**				
Sex (reference: female)	-2.00	(-3.04,	-0.96)	< 0.001
Age	0.02	(-0.07,	0.11)	0.673
Body mass index	0.01	(-0.15,	0.16)	0.937
Chronic illness (reference: none)	1.35	(0.28,	2.42)	0.014
**Rhythm Regularity**				
Intradaily stability	-0.46	(-5.46,	4.54)	0.856
Interdaily variability	1.61	(-2.13,	5.35)	0.395
Relative amplitude	-14.23	(-22.21,	-6.26)	0.001
**Activity Counts**				
L5	-0.50	(-0.88,	-0.12)	0.011
M10	0.23	(-1.20,	1.65)	0.754
Acrophase in cosinor analysis	0.02	(-0.26,	0.31)	0.869
**MVPA Distribution**				
Intensity gradient	0.04	(-0.70,	0.79)	0.907
Power-law exponent α	-0.16	(-4.32,	4.00)	0.940

*Abbreviations*: BSRS-5 = 5-item Brief Symptom Rating Scale; L5 = Average activity during the least active 5-hour period, log-transformed; M10 = Average activity during the most active 10-hour period, log-transformed; MVPA = Moderate to Vigorous Physical Activity

## Data Availability

The data that support the findings of this study are available from the corresponding author, WJC (email: s871056@nhri.edu.tw), upon reasonable request.
